# Supplementary material for: Asymmetric electric field screening in van der Waals heterostructures
Source: Nat Commun. 2018 Mar 28;9:1271. doi: 10.1038/s41467-018-03592-3 (PMC5871878; doi:10.1038/s41467-018-03592-3)
Supplement: Supplementary file 1 — Supplementary Information [file 41467_2018_3592_MOESM1_ESM.pdf]

Supplementary Information

Asymmetric Electric Field Screening in van der Waals Heterostructures

Lu Hua Li,[1] Tian Tian[2], Qiran Cai,[1] Chih-Jen Shih[2], and Elton J. G. Santos[3]*

[1]*Institute for Frontier Materials, Deakin University, Geelong Waurn Ponds Campus, Victoria 3216, Australia*

[2]*Institute for Chemical and Bioengineering, ETH Zürich, 8093 Zürich, Switzerland*

[3]*School of Mathematics and Physics, Queen's University Belfast, Belfast BT7 1NN, United Kingdom*

*Author to whom all correspondence should be addressed. Email: e.santos@qub.ac.uk

Supplementary Note 1: Qualitative analysis on the EFM result

We tried to establish a qualitative agreement between the simulation and EFM experiment using very rough approximations. The most widely used simplified model to estimate EFM interactions is by Hudlet et al.[1] When the sample is completely flat and the cantilever tip has a cone shape, as well as the lever interaction is ignored, the electrostatic force can be simplified as:

$$F = \pi\varepsilon\left[\frac{R^2(1-\sin\theta_0)}{z[z+R(1-\sin\theta_0)]} + \frac{1}{\left[\ln\tan\left(\frac{\theta_0}{2}\right)\right]^2}\left(\ln\frac{z+R(1-\sin\theta_0)}{H} - 1 + \frac{R\frac{\cos^2\theta_0}{\sin\theta_0}}{z+R(1-\sin\theta_0)}\right)\right](V+V_{CPD})^2 \qquad (1)$$

where F is the electrostatic force felt by the conductive cantilever tip during measurements; ε is

the permittivity of the gap between the tip and sample; R is the radius of the apex of the tip; θ_0 is the angle between the tip apex and the tip cone; z is the distance between the tip apex and the flat sample; H is the height of the tip cone. V is the voltage applied to the substrate; V_{CPD} is the contact potential difference. The geometric parameters of the tip used in the calculation can be found in Supplementary Figure 1.

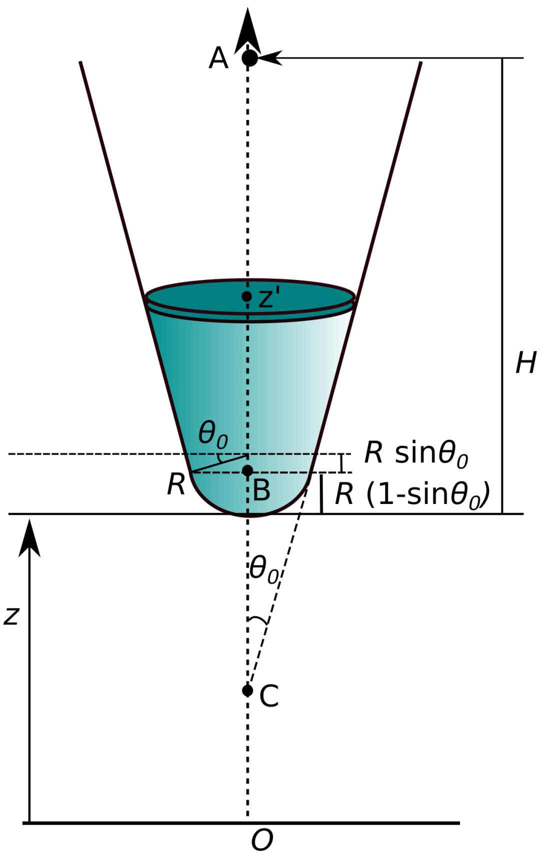

Supplementary Figure 1: Schematic of the EFM tip. Geometric parameters of the cantilever tip used in the simplified model to qualitatively analyze EFM interactions[1].

Combining Eq.1, 2 and Supplementary Equation 1, we can calculate the electric susceptibility

based on the EFM results. The permittivity of the gap between the tip and the Au substrate is estimated by:

$$\varepsilon = \kappa\varepsilon_0 = \frac{(t_1+t_2)\kappa_1\kappa_2}{t_1\kappa_2+t_2\kappa_1}\varepsilon_0 \qquad (2)$$

where κ is the relative permittivity of the gap; κ_1 and κ_2 are the relative permittivity of the heterostructure and air (κ_2=1); t_1 and t_2 are the thickness of the heterostructure and air between the tip and the sample. The thickness of 3L MoS2/4L Graphene is 3.29nm; the tip/sample distance is 8nm. However, due to the many unknown parameters in Supplementary Equation 1, we could not directly calculate the electric susceptibility of the heterostructures; instead, we use the difference between the left and right side of the asymmetric parabola (Figure 2) for calculating the change of the electric susceptibility of 3L MoS_2/4L graphene, given κ_1=1.2 when the electric field goes in graphene and comes out from MoS_2. The qualitative result as illustrated in Supplementary Figure 2 shows that there is a gradual increase in the electric susceptibility of the heterostructure as the bias points towards MoS_2 and more voltage is applied in EFM. The high-dispersion of points near 0 V is due to small tip voltage (V), giving rise to bad signal-to-noise ratio in electrostatic force and hence phase change.

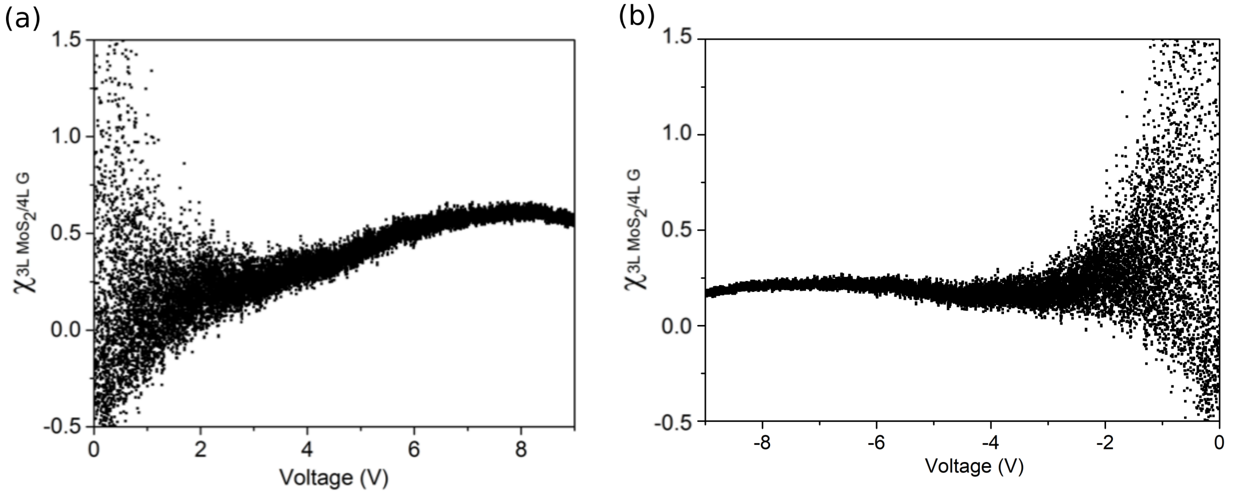

Supplementary Figure 2: Electric susceptibility from EFM data. Estimated electric susceptibility χ of 3L MoS_2/4L Graphene under (a) positive and (b) negative voltages. Positive (negative) voltages go towards MoS_2 (Graphene) in EFM.

Note that the qualitative analysis is based on many rough approximations. For example, geometry has a critical effect on the EFM interactions, and in our experiment the cantilever tip has a non-conical or asymmetric shape[2]. However, there has been no simplified model suitable for such tip geometry.

Supplementary Note 2: Interlayer distance between graphene and MoS_2 sheets

We performed simulations using different van der Waals (vdW) density functional (DF) to clarify whether different approaches on the exchange potential would give different results on the interlayer distance between graphene and MoS_2 sheets. We have used the original Dion et al. vdW-DF[3] (DRSLL), with different modifications on the exchange part. They are those modified by K. Berland and P. Hyldgaard[4] (BH), by V. R. Cooper[5] (C09), and by J. Klimes, D. R. Bowler and A. Michaelides[6] (KBM). Supplementary Table 1 summarizes the main results obtained. We

found that despite the vdW-functional utilized minor modifications are noted at the interlayer distance between graphene and MoS_2 sheets. All three modifications on the exchange functional give results at the same magnitude, which differ by around 2% of the original DRSLL functional. Indeed, these values compare well to the recent literature also using similar setup and functionals[7-8]. This indicates that the choice of the vdW functional is not so critical on the description of the graphene-MoS_2 interface[9].

vdW-DF	DRSLL	BH	C09	KBM
$d_{G\text{-}MoS2}$(Å)	3.37	3.30	3.29	3.29

Supplementary Table 1: Interlayer distance between graphene (G) and MoS_2 layers calculated using different van der Waals density functional (vdW-DF). The interlayer distance is taken from the carbon plane to the topmost S atom at the MoS_2 layer near to graphene.

We also estimate the effect of different interlayer distances on the results of the electrostatic model. We take the 1L Graphene/ 1L MoS_2 as an example, and analyze the charge density Q_G as a function of E_{ext}, as illustrated in Supplementary Figure 3. It is observed, that the choices of different vdW functionals have almost negligible influence on the results of the electrostatic model. Therefore, the conclusions using the electrostatic model remain valid, regardless of the vdW functionals used in the *ab initio* simulations.

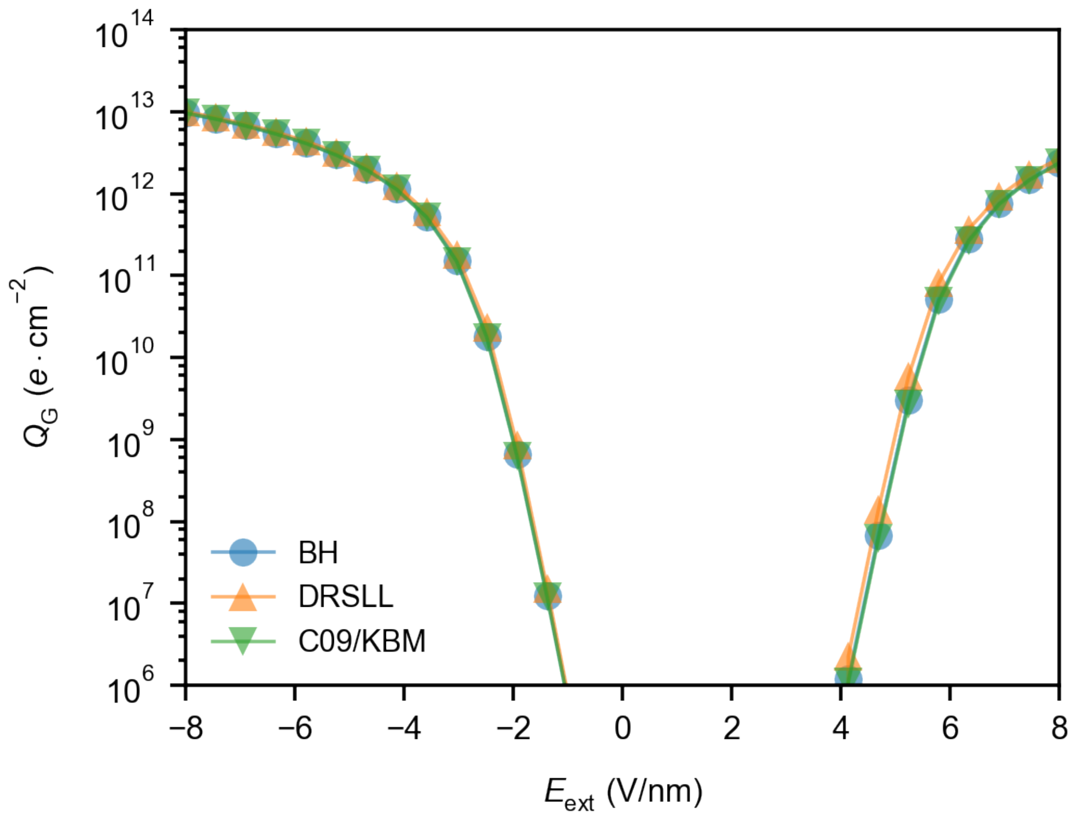

Supplementary Figure 3: Charge density calculated using different vdW functionals.

Charge density Q_G as a function of E_{ext} of a 1L Graphene/ 1L MoS_2 configuration, with the interlayer distances estimated from different vdW functionals (DRSLL, BH, C09 and KBM). The different choices of vdW functionals show almost negligible influence on the electrostatic model.

Supplementary References

1. Hudlet, S., Saint Jean, M., Guthmann, C., Berger, J. Evaluation of the capacitive force between an atomic force microscopy tip and a metallic surface. *Eur. Phys. J. B* **2**, 5-10 (1998).

2. https://afmprobes.asylumresearch.com

3. Dion, D., Rydberg, H., Schröder, E., Langreth, D. C., Lundqvist, B. I. Van der Waals Density Functional for General Geometries. *Phys. Rev. Lett.* **92**, 246401 (2004).

4. Berland, K, Hyldgaard, P. Exchange Functional that Tests the Robustness of the Plasmon Description of the van der Waals Density Functional. *Phys. Rev. B* **89**, 035412 (2014).

5. Cooper, V. R. Van der Waals density functional: An Appropriate Exchange Functional. *Phys. Rev. B* **81**, 161104(R) (2010).

6. Klimes, J., Bowler, D. R., Michealides, A. Chemical Accuracy for the van der Waals Density Functional. *J. Phys. Condens. Matter.* **22**, 022201 (2010).

7. Ma, Y., Dai, Y., Guo, M., Niu, C., Huang, B. Graphene adhesion on MoS_2 monolayer: An ab initio study. *Nanoscale* **3**, 3883-3888 (2011).

8. Hu, W., Wang, T., Zhang, R., Yang, J. Effects of interlayer coupling and electric fields on the electronic structures of graphene and MoS_2 heterobilayers. *J. Mat. Chem. C* **4**, 1776-1781 (2016).

9. Liu, X., Li, Z. Electric Field and Strain Effect on Graphene-MoS_2 Hybrid Structure: Ab Initio Calculations. *J. Phys. Chem. Lett.* **6**, 3269-3275 (2015).
